# Liquid biopsies and minimal residual disease in myeloid malignancies

**DOI:** 10.3389/fonc.2023.1164017

**Published:** 2023-05-05

**Authors:** Sabine Allam, Kristina Nasr, Farhan Khalid, Zunairah Shah, Mahammed Ziauddin Khan Suheb, Sana Mulla, Sindhu Vikash, Maroun Bou Zerdan, Faiz Anwer, Chakra P. Chaulagain

**Affiliations:** ^1^ Department of Medicine and Medical Sciences, University of Balamand, Dekwaneh, Lebanon; ^2^ Department of Internal Medicine, Monmouth Medical Center, Long Branch, NJ, United States; ^3^ Department of Internal Medicine, Weiss Memorial Hospital, Chicago, IL, United States; ^4^ Department of Internal Medicine, St. Luke’s hospital, Milwaukee, WI, United States; ^5^ Department of Internal Medicine, St Mary’s Medical Center, Apple Valley, CA, United States; ^6^ Department of Medicine, Jacobi Medical center/AECOM Bronx, Bronx, NY, United States; ^7^ Department of Internal Medicine, SUNY Upstate Medical University, New York, NY, United States; ^8^ Department of Hematology and Oncology, Taussig Cancer Center, Cleveland Clinic, Cleveland, OH, United States; ^9^ Department of Hematology and Oncology, Maroone Cancer Center, Cleveland Clinic Florida, Weston, FL, United States

**Keywords:** liquid biopsies, minimal residual disease (MRD), acute myeloid leukemia, acute myeloid leukemia (AML), myelodysplastic syndromes (MDS), myeloid malignancies, blood biomarkers, artificial intelligence - AI

## Abstract

Minimal residual disease (MRD) assessment through blood component sampling by liquid biopsies (LBs) is increasingly being investigated in myeloid malignancies. Blood components then undergo molecular analysis by flow cytometry or sequencing techniques and can be used as a powerful tool for prognostic and predictive purposes in myeloid malignancies. There is evidence and more is evolving about the quantification and identification of cell-based and gene-based biomarkers in myeloid malignancies to monitor treatment response. MRD based acute myeloid leukemia protocol and clinical trials are currently incorporating LB testing and preliminary results are encouraging for potential widespread use in clinic in the near future. MRD monitoring using LBs are not standard in myelodysplastic syndrome (MDS) but this is an area of active investigation. In the future, LBs can replace more invasive techniques such as bone marrow biopsies. However, the routine clinical application of these markers continues to be an issue due to lack of standardization and limited number of studies investigating their specificities. Integrating artificial intelligence (AI) could help simplify the complex interpretation of molecular testing and reduce errors related to operator dependency. Though the field is rapidly evolving, the applicability of MRD testing using LB is mostly limited to research setting at this time due to the need for validation, regulatory approval, payer coverage, and cost issues. This review focuses on the types of biomarkers, most recent research exploring MRD and LB in myeloid malignancies, ongoing clinical trials, and the future of LB in the setting of AI.

## 1 Introduction

Liquid biopsy (LB) probes the genomic profile of the tumor using peripheral blood (PB) components. Various tumor constituents can be extracted from the blood which can help in developing individualized therapies, monitoring tumor progression, monitoring treatment responses, and quantifying minimal residual disease (MRD). This clinical tool has the benefit of being non-invasive. It can provide valuable information regarding the burden of the disease, the tumor’s heterogeneity, and evolution (1).

LB is composed of different biological matrices such as circulating tumor cells (CTCs), cell-free circulating nucleic acids (DNA, mRNA, micro-RNA, or non-coding RNA), “tumor-educated platelets” (TEPs), and exosomes. CTCs are shed into the bloodstream early in tumorigenesis (2). CTCs thus represent a helpful marker in early screening, diagnosis, and relapse detection (3). CTCs can be isolated from the blood by using physical and biological properties such as tumor-specific surface antigens (4). Comparing genomic sequencing of individual CTCs with genomes of the primary tumor, can provide new insight and aid in the detection of early metastasis (3). Several gene mutations like *EGFR*, *KRAS*, and *HER 2* can also be analyzed from the CTCs guiding the development of targeted therapy and improving outcomes (5). The amount of CTCs identified has been linked to the efficacy of treatment and the overall survival of patients (6).

Another component of LBs includes circulating RNAs (cRNAs) which were first identified in patients with solid malignancies in late 1990s (7). Blood-based RNA profiling can provide pivotal information regarding tumor-specific gene expression and epigenetic alterations carried by the tumor. Micro RNAs (mi-RNAs) are the most common cRNAs and display extreme stability because they are carried by plasma proteins, exosomes apoptotic bodies, and tumor-educated platelets (TEP) (4). Their genomic landscape seems to correlate with the tumor they originate from (8). Exosomes are microvesicles released from the cells via exocytosis when intracellular vesicles fuse with the cell membrane (4). Exosomes can be released from neoplastic cells and contain molecules that carry vital information about endothelial gene fusion as well as gene expression profiling (9). Exosomes contain a large amount of RNA which can be advantageous in tumor profiling where other LB components can be of low yield (10).

Similarly, circulating tumor DNA (ctDNA) recently came out as another entity obtained through a noninvasive procedure for the identification of tumor evolution, resistance, and heterogeneity during cancer treatment. ctDNA refers to the fragmented cell free DNA (cfDNA) found in the plasma of cancer patients. In healthy individuals, apoptotic cells appear to be the primary source of cfDNA. While the exact reason behind the release of ctDNA is not clear, the length of the DNA fragments may provide some clues as to its source [29 in sab Julien]. As cancer cells have a higher turnover rate, there is a significant increase in cell free DNA in cancer patients. The latter is released from primary tumors, metastasic lesion, and CTCs (11). ctDNA is usually found in a low percentage (0.01-1%) of the total cfDNA and depends on the type, stage, location, and vascularization of the tumor (review nature) (12). ctDNA contains specific mutations that are unique to the tumor which account for dynamic changes making it more helpful when tracking tumor progression (13). It is particularly effective in the early diagnosis of relapse after patients’ complete treatment, as higher levels of ctDNA are correlated with a higher risk of early relapse. Assessing genetic mutations in ctDNA helps in formulating personalized therapies. ctDNA analysis yields insights into the intratumoral diversity and clonal evolution (14). This can be used in assessing the progress of the disease to detect response or failure to ongoing treatments which is vital for clinical decision-making and appropriate management (15).

Other examples of LBs playing a pivotal role in monitoring tumor progression and tumor development include tumor educated platelets (TEP). Although platelets are anucleated, they possess an RNA profile that can be altered following tumor interaction (16). This can make TEP helpful in monitoring tumor growth and assessing treatment responses (17).

Using LB for cancer surveillance presents an opportunity to study various aspects of the disease, including early detection, identification of minimal residual disease, relapse interception, and evaluation of treatment efficacy.

## 2 Minimal residual disease assessment using LB in myeloid malignancies

Minimal residual disease or measurable residual disease (MRD) refers to the persistence malignant cells in patient who receive seemingly successful initial treatment (18). This MRD can potentially lead to metastatic relapse at remote sites. MRD detection and monitoring are already widely used in hematological malignancies but remain difficult to apply in solid tumors due to the low blood levels of CTCs that make sampling a challenge (15). MRD serves as an important marker in myeloid malignancies and is beneficial in prognostication, disease monitoring, detecting early relapse, guiding post-remission treatment, and measuring treatment efficacy (19). While different techniques have been developed for the assessment of MRD in myeloid malignancies (see **Table 1**), MRD test result indicates the presence “MRD positive” or absence “MRD negative” of detectable disease above certain thresholds, which may differ depending on the assay and laboratory used (19). Flow cytometry analyses the protein expression on the cell surface or in the cytoplasm by use of fluorescently labeled antibodies and can determine the immunophenotype of cell lineages (20). Flow cytometry is suitable for MRD detection in Acute Myelocytic Leukemia (AML) and Myelodysplastic syndromes (MDS) (21). Quantitative Reverse Transcriptase Polymerase Chain Reaction (RT-qPCR) comprises converting the mRNA extracted from the specimen to cDNA through reverse transcription and then quantifying the cDNA via PCR. *ABL1* for example is the most common gene expressed in chronic myeloid leukemia white cells and PCR amplification followed by testing for overexpression of transcripts makes RT-qPCR one of the most sensitive methods for MRD detection (22). With recent advancements in liquid biopsies, Next Generation Sequencing (NGS) is currently being used to monitor response after therapy for myeloid malignancies, especially AML (23). In NGS, DNA fragments from regions of interest are amplified by PCR and sequences are compared with reference sequences. NGS is particularly advantageous in identifying mutations in the exons like *NPM1* for AML (24).

**Table 1**, in **Supplementary Material**, portrays some of the ongoing studies utilizing liquid biopsies in myeloid malignancies.

**Table 1:** Minimal Residual Disease (MRD) monitoring using liquid biopsies in myeloid malignancies (20, 25).

Method	APL	AML	MDS	MPN	CML
**Flow cytometry**	No	Yes	Yes	No	No
**Molecular methods (RT-qPCR)**	Yes	Yes	No	No	Yes
**NGS methods**	No	Yes	No	No	No

Minimal Residual Disease (MRD), Acute Promyelocytic Leukemia (APL), Acute Myelocytic Leukemia (AML), Myelodysplastic syndrome (MDS), Myeloproliferative Neoplasm (MPN), Chronic Myelocytic Leukemia (CML), Quantitative Reverse Transcriptase Polymerase Chain Reaction (RT-qPCR), Next Generation Sequencing (NGS).

s

### 2.1 Acute myeloid leukemia

Acute myeloid leukemia (AML) is a clonal neoplastic disease of the myeloid lineage precursor stem cell with a heterogenous character and dismal survival (26). Despite efforts into improving and expanding therapy options, AML diagnosis remains a challenge. Therefore, the accurate diagnosis and categorization of AML are crucial prior to treatment initiation. The treatment starts with induction chemotherapy, achieving cytologic complete remission (CR) in almost 70% of patients (27). Half of these patients relapse within 6 months in the absence of further treatment (27). It was found that a CR state does not always equate to cured disease. Residual cancer cells may continue to exist at levels below the threshold for cytomorphological detection and ultimately lead to relapse. The minimal/measurable residual disease (MRD) refers to the persistence of malignant blasts at levels undetectable by conventional morphologic methods (1:1000-1:106 white blood cells) (28). Currently, MRD is detected in AML patients using multiplanar flow cytometry (MFC) and other molecular techniques. MFC-MRD measures the load of leukemic blast cells and is referred to as the leukemia-associated immunophenotypes (LAIPs) (18). Real-time quantitative PCR (RT-qPCR) is commonly used for molecular PCR (29). Digital droplet PCR (ddPCR) is now emerging as a more sensitive and specific tool in recent studies (25, 29, 30).

LB using PB have emerged as an attractive option for detecting MRD. It allows monitoring the dynamics of a specific treatment protocol in an environment of constantly changing genomic aberrations. With longitudinal follow-up and ease of sampling, the clonal evolution of AML is identified and classified into its leukemic subtype. This allows for potential new drug targets and more individualized therapy for post-remission strategies or relapse, causing less treatment-related toxicities and better outcomes (31, 32). PB contains different biological cells and can be divided into cell-based and gene-based biomarkers.

CTCs, as a method of detection in LB, have a promising potential. Recently, the quantification of CTCs has been incorporated into the MRD AML protocol (33). In a prospective AML study monitoring MRD after CR, PB samples were used as an alternate to bone marrow (BM) aspirates and showed concordance in the CTC quantification derived from both sources. The MRD status in the PB group was found to significantly affect relapse-free survival both post-induction and post-consolidation: of all MRD + patients on PB, 77% had a relapse after induction (p=0.0002) and 82% had a relapse after consolidation (p=0.00006). The study concluded that PB could be integrated into MRD detection protocols in patients with AML, and prognostic information can be obtained at the end of PB MRD consolidation therapy (34).

Other cell-based biomarkers are endothelial cells that detach from the blood vessel wall as a result of physical injury or inflammatory, autoimmune, or cardiovascular diseases (35, 36). In AML, a higher concentration of circulating endothelial cells (CECs) in the PB correlates with disease severity and chemotherapeutic response. This principle can be used to evaluate MRD in AML patients (37). CECs influence AML progression through mechanisms that are not completely understood. Liesveld et al. reported that co-culturing endothelial cells with leukemic cells prevented cytarabine-mediated apoptosis, showing an increase in the proliferation and survival of the leukemic cells (38). Endothelial cells secrete cytokines and adhesion molecules that assist with chemoresistance and leukemic blast survival (38). Hence, CECs can serve as useful indicator for assessing post-treatment remission and evaluating the effectiveness of allogeneic stem cell transplant (39).

Moving on to other components of LB, microRNAs (miRNAs) are gaining focus as easily detectable gene-based biomarkers, owing to their higher sensitivity and stability in biological fluids (up to 4 days at room temperature) (see **Table 2**). In addition, the quantification of extracellular miRNAs is becoming easier with recourse to newer technologies (e.g., NGS) that detect nucleic acids with high sensitivity. Various studies describe different miRNAs as diagnostic and prognostic biomarkers. As such, miRNA was useful when differentiating AML from healthy controls: higher expression levels of miR-155-5p and miR-181b-5p and lower levels of let-7d, miR-150, miR-339, and miR-342 were characteristic of AML patients (46). Similarly, elevated miR-150 and miR-342 expression after induction chemotherapy was linked to CR (45). Concerning prognosis, expression of miR-10-5p was higher in relapsed patients. Fang et al., in their study, showed that high serum extracellular miR-10-5p expression was associated with disease aggressiveness, indicating a poor prognosis (41). Additionally, Guo et al. described biomarker levels of miR-203 as necessary for both diagnosis and prognosis. In this study, low serum miR-203 was diagnostic of AML patient and was associated with worse outcomes as measured by overall survival (OS) and relapse-free survival (RFS) (47).

**Table 2:** miRNAs markers in hematological malignancies.

mRNAs marker	Application	Specimen source	References
miR-203	D & P	Serum	(40)
miR-10b	D & P	Exosomes	(41)
miR-155	P	Blood	(42)
miR-210	P	Serum	(43)
miR-10-5p	D & P	Serum	(44)
miR-150, miR-342	D & P	Plasma	(45)
miR-155-3p, miR-181-5p	D & P	Serum	(46)

D, diagnostic; P, Prognosis.

Another gene-based biomarker is cfDNA. The concept of cfDNA in AML was first reported in early 90s by Vasioukhin and Stroun’s who showed that cfDNA carried *N-RAS* point mutation in AML and MDS patients. Generally, the methods used to detect cfDNA are qRT-PCR, targeted next-generation sequencing (NGS), and mass spectroscopy. These techniques have a variant allelic frequency (VAF) as low as 0.01% with high sensitivity and specificity (48). The usefulness of cfDNA for detecting and monitoring MRD negativity in myeloid disorders has been displayed in different studies (see in **Table 3**). A study by Gao et al. analyzing PB samples from 60 AML patients and 30 healthy donors, showed significantly higher cfDNA concentrations and integrity in cancer patients. ctDNA integrity was reflective of MRD status and AML progression, demonstrating a decrease during CR and an increase upon relapse (55). Furthermore, Zhong et al. proved the utility of ctDNA in detecting monoclonal IGH and TCR rearrangement. While around 90% of patients had cfDNA without rearrangements directly after consolidation, all patients demonstrating cfDNA rearrangements relapsed on follow-up which confirmed that cfDNA MRD assessment is predictive of disease outcomes. In addition, the recurrence of these rearrangements in the PB cfDNA was observed about 1-3 months earlier than shown through BM biopsy. This highlighted once again the potential role of ctDNA as biomarker for MRD monitoring in AML patients indicating that ctDNA MRD monitoring could be equally or even possibly more informative than BM biopsy (52).

**Table 3:** Overview of various studies demonstrating the utility of ctDNA for molecular characterization, disease monitoring, and clinical application in acute myeloid leukemia.

Study	Year	Patients/controls	Method	Gene Target	Clinical application
Shao et al. (49)	2022	103/81	Targeted NGS	5hmC gene target	D & P
Short et al. (50)	2020	22	Targeted NGS	28 targets	D & P
Nakamura et al. (51)	2019	53	NGS, ddPCR	57 targets	D & P
Zhong et al. (52)	2018	235	qPCR	TCR gene rearrangement	P
Quan et al. (53)	2015	100	qRT-PCR	NPM1	D
Jiang et al. (54)	2012	66/100	Duplex real-time qPCR	–	D & P
Gao et al. (55)	2010	60/30	qPCR	ACTB	P
Mueller et al. (56)	2006	25	Spectrophotometry	–	D & P
Rogers et al. (57)	2004	45/30	Microsatellite markers	5q,7q,8,17p,20q	D
Vasioukhin et al. (58)	1994	10	Southern hybridization	N-RAS	D & P

D, diagnostic; P, Prognosis.

Over the years, the genomic profiling of cfDNA has helped identify the driver mutations and karyotypic anomalies in AML to monitor the disease and evaluate treatment outcomes. For example, Nakamura et al. highlighted the potential utility of ctDNA as prognostic tool in AML patients who have undergone allogeneic stem cell transplant. The researchers analyzed ctDNA in 81 patients with AML and found that ctDNA positivity post-transplant was associated with significantly lower rates of RFS and OS in these patients. Specifically, 2-year RFS rates were 29.6% for ctDNA-positive AML patients compared to 85.7% for ctDNA-negative AML patients (p < 0.001), while 2-year OS rates were 39.3% for ctDNA-positive AML patients compared to 85.7% for ctDNA-negative AML patients (p < 0.001) (51).

A recent study by Shao et al. investigated the potential of use cfDNA 5-hydroxymethylcytosine (5hmC) analysis as a biomarker for AML. The researchers analyzed cfDNA from 103 AML patients and 81 controls and developed a 5hmC diagnostic model that consisted of a 70 gene signature. 5hmC level of the 70 genes were significantly higher in AML patients (P < 0.001) and positively correlated with AML disease burden (49). Therefore, 5hmC analysis permitted real-time monitoring of the disease with high sensitivity as it had the ability to capture small amounts of ctDNA even before development of overt leukemia. 5hmC served both as a diagnostic and a prognostic model. The top 2 survival predictive 5hmC gene markers were *BMS1* and *GEMIN5* significantly associated with worse OS in AML patients (49). Similarly, Rausch et al. discussed the applicability of double drop-off digital droplet PCR (DDO-ddPCR) assays for gene mutation in *NPM1*, *IDH2*, and *NRAS* (59). These assays were then validated with NGS and ddPCR methods. High levels of concordance was seen between the DDO-ddPCR and conventional digital PCR when comparing sensitivity. Both methods monitored and detected genetic alterations in PB cfDNA with similar sensitivity. After testing in different clinical scenarios, cfDNA-based digital PCR was proven helpful for therapy monitoring, chemotherapy response evaluation early-on in induction phase, and identification of mutations (59). Furthermore, Short et al. compared the diagnostic accuracy of targeted NSG of cfDNA with BM in AML patients both at the time of diagnosis and post-remission. The researchers collected paired samples of cfDNA and BM from 22 patients and assessed VAF among 28 genes. They found that the diagnostic accuracy of cfDNA was comparable to that of BM as both sources were able to detect 19 out of 39 somatic mutations with high concordance (R^2 =^ 0.849) (50). Out of the 20 remaining mutations, 13% were detected using cfDNA and 38% using BM, hinting towards a possible complementary role of cfDNA and BM sequencing as cfDNA can catch clinically relevant mutations that are not detected in the BM. When the VAF was <10%, both cfDNA and BM samples missed small subclonal populations (50). The fact that some mutations were not detectable using tissue based NGS (43 in original doc) brings out limitations of NGS BM biopsy with failure rates of approximately 14% are recorded in large centers (44 In original doc). On the other hand, integrating cfDNA NGS testing has shown a significant increase in the detection of therapeutically targetable mutations, refining molecularly guided therapy. cfDNA has been extensively researched and confirmed as the most established and reliable liquid biopsy source (60).

The above-mentioned studies demonstrate outstanding potential for ctDNA in AML MRD assessment. However, various challenges exist. The International Society of liquid biopsy (ISLB) has reported various limitations associated with the cfDNA analysis (60). The logistics of sample collection and processing affect the sensitivity of cfDNA analysis. Hence, Avoiding WBC lysis and subsequent genomic DNA contamination is crucial to the appropriate analysis.

To overcome this issue, Shin et al. suggested that the samples be processed within 4 hours or stored in tubes containing a stabilizer (61). Other potential limiting factors include the asynchronous growth of malignant leukemia clones at different sites which causes MRD subclones to be missed. In addition, the concurrent development of age-related clonal hematopoiesis of indeterminate potential (CHIP) mutations (e.g., *DNMT3A*, *TET2*, and *ASXL1*) represents a source of false positives in cfDNA. However, a CHIP-filtering approach based on whole blood sequencing and cfDNA analysis with a sensitivity equivalent to ddPCR has been developed recently (62, 63).

The use of LBs in AML MRD is evolving in the clinical research fields. As part of their recommendations in 2022, the ELN advocate for the use of blood samples as part of MRD testing in AML. Various other ongoing projects or initiatives are looking into the development of the next generation of AML MRD tests. The myeloMATCH initiative planned for launch in early 2023 is a North American precision medicine master protocol initiative for the myeloid malignancies (64). They aim to incorporate MRD testing of blood and BM samples both in randomized clinical trials both as endpoints and as inclusion criteria for subsequent lines of therapy. Sequencing based techniques might as well overcome the problem of single point-snapshot if they become longitudinal home based tests partly owing to their feasibility and low reduced costs. Large population cohorts, like HARMONY and MEASURE, are needed to answer these questions (65).

In conclusion, the clinical application of these biomarkers remains a concern due to the need for more standardization and the lack of studies investigating the specificity of the biomarkers. The question remains on the standardization of advancing technology and making it widely accessible. The ELN AML MRD working group is probably a crucial initial step. A common consensus by different working groups will need to be implemented to benefit all AML patients.

### 2.2 Myelodysplastic syndromes

MDS are a group of hematopoietic stem cell disorders with a vast spectrum of genetic and biological features. Clinical features of MDS include cytopenia and a potential risk for advancement to AML (66). For optimal patient management of hematological malignancies, it is essential to monitor response to treatment. More precise techniques are being developed to analyze the MRD status and cfDNA. Alternative approaches now detect increased CECs and provide insight into metabolic heterogeneity. DREAMing (Discrimination of Rare EpiAlleles by Melt) utilizes semi-limiting dilution and precise melt curve analysis for the evaluation of epigenetic heterogeneity (18, 67). There have been recent developments in the use of LBs for MRD monitoring despite several obstacles relating to the standardization of such techniques (18). **Table 4** demonstrates an overview of the studies mentioned below investigating the use of liquid biopsies in MDS potential of MRD assessment.

**Table 4:** Overview of studies demonstrating the use of Liquid Biopsy components for molecular characterization and monitoring of myelodysplastic syndromes.

Study	Year	Sample	Target	Molecular Technique Used	Goal of Study
Rodrigues et al. (63)	2022	AA: n=25 MDS: n=27 Healthy cohort: n=107	cfDNA	Commercial cfDNA assay	Compare cfDNA to matched cells in detecting clonal hematopoiesis
Nakamura et al. (64)	2019	AML: n=37 MDS: n=14	ctDNA	NGS and ddPCR	Identify patients at risk of relapse post-alloSCT based on ctDNA status
Waterhouse et al. (65)	2022	AML: n=48 MDS: n=4 MPN: n=7 CMML: n=2 AA: n=1	cfDNA	NGS	Identify the utility of cfDNA in monitoring MRD and MC in patients who underwent alloSCT
Gisbert et al. (66)	2022	MDS: n=70	cfDNA	NGS Chromosomal microarray	Assess the use of cytogenetic alterations in cfDNA to monitor patients with MDS
Andrew et al. (67)	2022	2821 samples from individuals with lymphoid and myeloid malignancies	cfDNA	NGS	Assess the reliability of NGS in detecting chromosomal structural abnormalities.
Giudice et al. (68)	2018	Healthy control: n=52 MDS: n=40 AA: n=70	Exosomal microRNA	PCR	Investigate plasma exosomal miRNAs as potential biomarkers of AA and MDS
Cerisoli et al. (69)	2020	MDS: n=34 CMML: n=5 AML: n=12 healthy controls: n=22	Exosomes	NTA Flow cytometry Quantative real time PCR	Isolate and characterize exosome phenotypically and isolate their contents

AA, aplastic anemia; AML, acute myeloid leukemia; alloSCT, Allogeneic hematopoietic stem cell transplantation; BM, bone marrow; cfDNA, cell free DNA; ctDNA, circulating tumor DNA; ddPCR, droplet digital PCR; MC, mixed chimerism; MDS, myelodysplastic syndrome; MRD, minimal residual disease; NGS, next generation sequencing; NTA, Nanoparticle Tracking Analysis; PB, peripheral blood; PCR, polymerase chain reaction.

In a study by Rodrigues et al., 2022, the PB cfDNA samples of patients with hematologic malignancies were screened via commercial cfDNA assay for somatic mutations and compared to DNA sequencing in matched blood cells. The study included those with aplastic anemia (AA; n = 25), MDS (n = 27), and a healthy cohort (n = 107) (68). According to the results, in healthy individuals and diseases with low allele burdens, proof of clonal hematopoiesis by screening cfDNA and comparing it to DNA of matched blood cells was poor. This was evident when VAF <10% was observed in the healthy and AA groups but not the MDS one. In order to overcome this obstacle and increase the accuracy for detecting low burden alleles, ultra-sensitive assays with robust sequencing coverage and error-correction methodology will be essential (68).

Nakamura et al., 2019 studied the role of ctDNA in monitoring relapse after an alloSCT. Retrospective data from 53 patients with AML/MDS was collected from tumors and available matched serum samples at diagnosis, 1 month, and 3 months post-alloSCT. NGS was used to identify driver mutations in 51 patients, and at least one personalized digital PCR assay per case was designed (69). Excellent correlations with VAFs were exhibited by diagnostic ctDNA and matched tumor DNA. After the allogeneic transplantation, increased ctDNA levels between months one and three were correlated with a higher 3-year cumulative incidence of relapse rates (69).

A similar study by Waterhouse et al. evaluated the clinical utility of cfDNA to assess MRD status and molecular chimerism (MC) in 62 patients who underwent alloSCT in order to detect earlier relapse. The study reported a significantly higher percentage (p<0.001) of recipient-derived cfDNA in patients who relapsed after alloHSCT than in those who were in complete remission. After applying receiver operating characteristic (ROC) curve, the optimal threshold of recipient derived cfDNA used to distinguish relapse from non-relapse was 18%. The study also illustrated that there was a statistically significant difference (p<0.001) in MC status of cfDNA between patients who are in relapse versus those in complete remission after alloHCT (70). Another important observation was that increasing MC and MRD positivity could be detected earlier in cfDNA when compared with PBMCs in a subset of patients. Even though this time difference was not statistically significant, earlier relapse detection enabled prompt clinical intervention leading to possible improved allo-HSCT outcome) (70). To note, the findings of this study should be further supported by other studies with a longer follow-up period and larger sample size.

In addition, Gisbert et al. assessed the molecular and cytogenetic profile of 70 patients with MDS. Compared to healthy controls, the amount of cfDNA collected in MDS patients was significantly higher (P=0.023) with a median of 58.4 ng/ml versus 32.4 ng/ml in the former. cfDNA concentration was also significantly higher in lower-risk patients with MDS than that in the healthy control group (P= .023) (71). The study also showed a comparable mutational profile when sequencing BM DNA and cfDNA with 92.1% concordance. The VAFs of both sample types were significantly correlated (P<0.001). Certain mutations were better represented in cfDNA sequencing library than in BM libraries such as *SF3B1*. The study also compared the use of NGS versus chromosomal microarray in identifying cytogenetic alterations of cfDNA and BM cell DNA. Both techniques showed high concordance. However, both techniques showed lower sensitivity when compared to fluorescence *in situ* hybridization and karyotyping (71).

Another study by Andrew et al. supported the use of LBs for early diagnosis and monitoring of patients with myeloid neoplasms. The study assessed the role of targeted NGS in identifying cytological variations of cfDNA in a sample of 2821 patients with myeloid and lymphoid malignancies. cfDNA PB samples were taken. 54.5% of the patients showed the presence of mutations consistent with neoplastic clones in the circulation (72). Out of the 54.5%, 41% showed abnormalities associated with lymphoid neoplasms while 59% showed abnormalities associated with myeloid neoplasms. In 89 AML or MDS patients stratified into intermediate, poor, or complex risk based on karyotype, there was a 100% concordance rate between cytologic and cfDNA specimens. Compared to BM samples, myeloid cfDNA using NGS samples accurately detected chromosomal gain and loss but were unable to diagnose fusion abnormalities (72).

Giudice et al. evaluated possible diagnostic and prognostic values of exosomal microRNAs in patients with AA and MDS. miRNAs are a marker of intracellular function including proliferation, metabolism, and cell survival. In a discovery cohort (n=42), 372 miRNAs were first screened. A customized PCR plate was then constructed, and 42 microRNAs were analyzed in a validation cohort of 99 patients. In AA and MDS patients before and after 6 months of immunosuppressive therapy, the study detected a correlation between miRNA level and clinical parameters (hemoglobin, WBC, platelet count, absolute neutrophil count, absolute lymphocyte count, lactate dehydrogenase) and progression-free survival. In MDS patients, 21 exosomal miRNAs displayed a strong association with the disease. Some of the miRNAs to note, miR-1180-3p showed positive correlation with hemoglobin level (r=0.483, P=0.036) and WBC count (r=0.561, P=0.013). While *miR-3200-3p* (r=0.963, P=0.002), *miR-196b-5p* (r=0.485, P=0.035), *miR-378i* (r=0.498, P=0.030), and *miR-1260a* (r=0.495, P=0.037) only to WBC count. However, no correlation was found between platelet count and miRNAs. Hence this study has successfully identified several miRNAs markers in SAA and MDS that can be used as candidate biomarkers of responsiveness to immunosuppressive therapy (73).

Another study by Cerisoli et al. isolated and characterized exosomes in MDS patients based on phenotype by analyzing their size and surface markers density (74). Whole blood was collected from patients with MDS (n=34), CMML (n=5), AML (n=12), and healthy controls (n=22). In MDS patients, a strong correlation with increased fluorescence intensity of CD34 was seen as compared to the healthy cohort. MDS samples showed downregulation of *miR-181a, miR-146a, and miR-155miR-16, miR-17, miR-20a, miR21, and miR-126* compared to the healthy cohort. This study further validates the use of exosomes as a biomarker for diagnosis and prognosis of MDS (74).

### 2.3 Myeloproliferative neoplasms

MPN are clonal hematopoietic stem cell (HSC) disorders characterized by the proliferation of one or more myeloid lineages. The four classic MPNs are chronic myeloid leukemia (CML), polycythemia Vera (PV), essential thrombocythemia (ET), and primary myelofibrosis (PMF). The major complications of the MPNs include thrombosis, bleeding, leukocytosis, splenomegaly, microcirculatory symptoms, pruritus, evolution to AML, MDS, and a fibrotic phase of the disease (75, 76). AlloHCT prolongs survival and can help cure the disease in patients with higher-risk primary myelofibrosis. The goal of available treatments in both PV and ET is to inhibit thrombo-hemorrhagic complications as medications have not been shown to improve survival or prevent complications (77). **Table 5** summarizes some of the most recent studies investigating the use of liquid biopsies in MPN and potential of MRD assessment.

**Table 5:** Overview of the most recent studies demonstrating the use of liquid biopsy components in the investigation of myeloproliferative malignancies.

Study	Year	Sample	Target	Molecular Technique Used	Goal of Study
Gisbert et al. (73)	2020	PV: n=33 ET: n=56 PMF: n=14 unclassifiable MPN: n=4	cfDNA	NGS	Investigate the use of cfDNA for molecular characterization of MPN
Zhang et al. (75)	2017	PV: n=20 ET: n=60 PMF: n=12	MP	Flow cytometry	Quantify MP variation in MPN patients and evaluate association with the *JAK2V617F* mutation and with thrombosis and splenomegaly
Baron et al. (78)	2019	MF: n = 61 ET: n = 20 Healthy control: n=20	MV	Flow cytometry	Investigate the role of MVs as biomarkers of malignancy in MPN
Ahadon et al. (79)	2018	PV: n=15 Healthy control: n=15	MP	Flow cytometry	Determine plasma derived microparticles in PV patients.
Mata et al. (80)	2022	histiocytic neoplasms: n=34 MDS: n=23 MPN: n=15 AML: n=11	ctDNA	NGS	Investigate the use of LB in the molecular characterization of myeloid malignancies using ctDNA as a biomarker
Mata et al (81)	2022	MPN: n=22 MDS: n=27 AML: n=10 Other hematologic malignancies: n= 212	ctDNA	NGS	Investigate the concordance between LB-based ctDNA and tissue-based approach in patients with HNs

CfDNA, cell free DNA; ctDNA, circulating tumor DNA; ET, essential thrombocythaemia; HN, hematologic neoplasms; LB, liquid biospy; MP, microparticle; MPN, myeloproliferative neoplasm; MV, microvesicle; NGS, next generation sequencing; PMF, Primary myelofibrosis; PV, polycythemia vera.

Genetic studies play a crucial role in accurately diagnosing and determining treatment plans, especially in patients with Philadelphia-negative MPNs. In a study by Garcia-Gisbert et al., the authors aimed to assess the accuracy and reliability of cfDNA compared to paired PB granulocyte DNA in identifying the molecular profile of MP patients. The study included 107 MPN patients: PV (n=33), ET (n=56), PMF (n=14), unclassifiable MPN (n=4) (82). In patients with PMF, MPL-mutated cases, or those with high molecular complexity a high concentration of cfDNA was detected in their plasma. This was evident when the amount of cfDNA measured per ml of plasma was significantly higher in patients with PMF (median 73.0 ng/ml) than in PV (median 17.4 ng/ml) or ET patients (median 14.3 ng/ml) (p< 0.001). Interestingly, it was found that in a median follow up of 15 months (range 1-60 months), patients suffering a thrombotic event at the time of diagnosis or during follow up (n = 10) had a significantly higher concentration of cfDNA with a median of 37.0 ng/ml of plasma compared to a group of patients without thrombotic events with a median of 17.0 ng/ml of plasma (p= 0.038) (82). The study also showed that there was an equivalent mutational profile between cfDNA and granulocyte DNA while inconsistencies were detected with variants at low VAF. Also, cfDNA may improve detection of certain mutations in MPNs. This was illustrated when the VAFs of mutations for *MPL*, *JAK2*, and *SRSF2* were detected higher in cfDNA than PB granulocyte DNA with a 66%, 20%, and 6% increase respectively. The study analyzed if cfDNA may be useful to monitor treatment response. It was found that in the follow-up period, the amount of *JAK2V617F* VAF in PV cases receiving hydroxycarbamide remained stable in both the granulocytes and cfDNA. However, a proportional decrease in the JAK2V617F VAF was observed in granulocytes and cfDNA of patients with ET being treated with interferon. Hence, cfDNA, when compared to PB granulocyte DNA, could be used as a fast, sensitive, and accurate strategy for identifying the molecular profile of MPN patients (82). In a study by Zhang et al. in 2017, blood of 92 patients with MPN was analyzed to identify the relation between circulating microparticles (MPs) and *JAK2V617F* mutation, thrombosis risk, and splenomegaly. Several types of microparticles were studied: endothelial MPs (EMPs), red blood cell MPs (RMPs), platelet-derived MPs (PMPs), and tissue factor MPs (TF+MPs). In PMF patients, EMPs, RMPs, PMPs, ad TF+MPs were detected in higher concentration than in healthy controls (p<0.01). The same was shown in patients with ET and PV (p<0.05). An elevated concentration of all types of MPs was associated with splenomegaly and a positive history of thrombosis (p<0.05).Also, PMPs were elevated in patients with *JAK2V617F* mutation compared to those without (p<0.05) (83, 84). This study illustrates the possible role of MPs in the pathogenesis of MPN and thrombosis. MPs may be used as a tool to assess disease severity and treatment response in MPNs.

Barone et al, investigated the use of extracellular microvesicles (MVs) as biomarkers of disease or malignancy in MPN. The study focused on several endpoints including: the profile of MVs in ET and MF, the relation between frequency of MVs and disease severity, the impact of inflammation on frequency of MVs in MF, and the effect of treatment with ruxolitinib on MVs in MF. Patients with MF (p < 0.01 and p < 0.001, respectively) and ET (p < 0.001 and p < 0.001 respectively) had significant higher levels of platelet-MVs but lower levels of megakaryocyte-MVs compared to controls. The level of platelet-MVs was significantly higher in JAK2 and CALR positive ET and MF patients when compared to ET and MF triple negative patients or control. Conversely, the level of megakaryocyte-MVs was significantly lower in JAK2 and CALR positive ET and MF patients when compared to ET and MF triple negative patients or control. According to the International Prognostic Scoring System (IPSS), MF subjects with high/intermediate-2 risk had elevated platelet-MVs but lower megakaryocyte-MVs compared to intermediate-1/low-risk (p < 0.05 and p < 0.01 respectively) or healthy subjects (p<0.001, for both). As for the inflammatory markers thrombopoietin and soluble P-selectin, the quantity of platelet-MVs was positively correlated to them (r = 0·51, p < 0·01; r = 0·36, p < 0·05, respectively). On the other hand, the percentage of megakaryocyte-MVs was negatively correlated to the level of interleukin-6 (r = −0·38; p < 0·05). Finally, after 6 months of ruxolitinib therapy, subjects who responded successfully to the treatment showed an increase in megakaryocyte-MVs (p<0.001), decrease in platelet-MVs (p<0.01), decrease in endothelial-MVs in spleen responders only (p<0.05), but no statistically significant decrease in monocyte-MVs. Hence this study further verifies the use of microvesicles as biomarkers to assess disease severity and response to treatment in MPN patients (78).

Another study by Ahadon et al. investigated the profile of microparticles in PV patients. In this study, PMPs positive for annexin V, CD61, and CD144 were detected by flow cytometry in patients with PV then compared to that in healthy controls. The results showed that PMPs were significantly elevated in PV patients compared to control (p<0.01). However, the level of EMPs were similar in both groups (p=0.43). The researchers also sought to identify a correlation between the number of thrombocytes and PMPs in PV patients. Even though levels of both markers were elevated, no linear correlation was registered. PMPs may be used as another biomarker for disease diagnosis, monitoring, and therapy (79).

A study by Mata et al. investigated the use of LB in the molecular characterization of myeloid malignancies using ctDNA as a biomarker. The patient pool included the following: histiocytic neoplasms (n=34), MDS (n=23), MPN (n=15), and AML (n=11). 147 pathogenic short variants and 6 rearrangements were identified including 61 variants with VAF<1%. This illustrates the improved sensitivity of this technique. This study illustrated that LB could identify clinically relevant genomic alterations even at subclonal levels. This further supports its role in monitoring myeloid malignancies and minimal residual disease testing (81).

Another study by Mata et al. investigated the use of a liquid biopsy-based NGS approach as alternative to tissue-based approach in the molecular profiling of lymphoid, plasma-cell, and myeloid malignancies. The median maximum somatic allele frequency (MSAF) among all cases was 7.3%, with higher median MSAFs in MPN (45.8%), AML (30.9%), and MDS (19.7%). LB detected specific genomic alterations across hematologic neoplasms. To illustrate: LB detected TP53, SF3B1, DNMT3A, TET2, and ASXL1 in MDS; JAK2 in MPNs; and FLT3, IDH2, and NPM1 in AML. Bone marrow aspirates and tissue specimens were collected from 42 patients. 31 variants out the 42 (positive percent agreement = 73.8%) variants detected by tissue-based approach were also detected by LB. Low ctDNA shed or heterogeneity related to disease course explained the non-concordance between the two approaches. Conversely, LB approach was able to detect more than one variant in 22 samples while tissue-based approach missed them. This may imply enhanced sensitivity of LB for detecting low-level disease clones unable to be detected by tissue specimen (80).

## 3 Role of artificial intelligence in MRD assessment using liquid biopsies

AI can play a role in MRD assessment by analyzing large amounts of data and identifying patterns that human reviewers may need help in detecting. Current manual interpretation suffers drawbacks such as interpreter idiosyncrasy and arduous labor. AI can help improve the accuracy and speed of MRD assessment, which can help guide treatment decisions and improve patient outcomes (85). As MRD assessment via liquid biopsy in myeloid malignancies remains under investigation, very little research exists on the use of AI tools in the assessment of MRD via information collected by liquid biopsies. Instead, we have included some of the most recent studies that investigated the role of AI in MRD assessment in myeloid malignancies. We also covered studies of AI and liquid biopsies in myeloid malignancies. **Table 6** provides a brief summary of the studies discussed below.

**Table 6:** Overview of different studies investigating the role of AI in Liquid Biopsies and in assessing MRD.

Study	Year	Sample	AI Tool Used	Goal
Patkar et al. (86)	2019	*NMP1* ^mut^ AML	ML	Create a ML-derived risk score to stratify patients with AML and correlate score with MRD
Li et al. (87)	2019	AML an MDS	DL	Create a DL-derived tool to classify patients with hematologic malignancies based on MRD status
Ni et al. (88)	2016	AML	SVM (ML)	Use of SVM to analyze MRD in AML patients using FC data
Ko et al. (77)	2018	AML and MDS	ML	Create a ML algorithm to detect MRD in AML and MDS patients using FC data
Guo et al. (82)	2022	Breast, prostate, hepatocellular cancers and melanoma	DL	Create a CTC tracer, a transfer learning-based algorithm, to transfer knowledge between different types of RNA-seq data of lesions and CTCs
Amor et al. (78)	2022	Breast, ovarian, and pancreatic cancer	AI nanoarray	Create a nanoarray that can detect viarious cancers through the production of volatile organic compounds collected by liquid biopsy

AI, Artificial Intelligence; AML, acute myeloid leukemia; CTC, Circulating Tumor Cell; DL, Deep Learning; ML, machine learning; MRD, Minimal Residual Disease; SVM, support vector machine.

As a branch of AI, machine learning (ML) has immense potential in interpreting large amounts of complex genomic data. ML can aid in interpreting the deluge of genomic data collected via NGS. ML can then create connections between several data sets in order to predict cancer susceptibility, recurrence, prognostication, and therapy. Based on this concept Patkar et al. used a ML approach to detect clinically significant genomic alterations in *NPM1mut* AML patients. Based on this data, a scoring model was developed to risk stratify patients. The study’s sample included 110 patients whose genomic profile was sequenced using 50-gene panel composed of 1066 single-molecule molecular inversion probes. Based on ROC analyses, cut-off values for VAFs of common mutations were decided to differentiate low risk groups from high-risk ones. The results showed that patients with high corrected *NPM1* VAF, low FLT3-ITD VAF, presence of *IDH2* mutation, absence of *DNMT3A R882* mutation, and type A *NPM1* mutation had a higher probability of survival. Based on the sum of the individual scores, a final score was created. The researchers also wanted to investigate if a correlation exists between their genetic score and post-induction multiparametric flow cytometry MRD (FCM-MRD). Out of the 99% of the patients who were in remission, FCM-MRD was detected in 27.1%. FCM-MRD showed inferior overall survival (p=0.007) and relapse free survival (p=0.01). These results illustrated a statistically significant correlation between post-induction FCM-MRD and the ML- derived genetic score created by the researchers (p=0.001). The results of this study show that this genetic scoring system can be used to identify NPM1mut AML patients at high risk of relapse (86). Despite the need for further validation of the cut-off values chosen, this study illustrates how AI-ML can help stratify patients efficiently and quickly in order to provide timely and optimal clinical interventions.

Another study by Li et al. explored the use of another AI tool, cytometric deep phenotype embedding, to assess the prognosis of patients with AML and MDS. The current gold standard in clinical practice is FC requiring highly trained physicians and lengthy manual interpretation. This study explores the potential of an automated classification of AML and MDS patients using MRD status via an algorithm based on learning a deep phenotype representation from data collected from FC samples of 2000 patients. The researchers incorporated cell-level autoencoder with specimen-level latent Fisher-scoring vectorization into cytometric deep embedding system. The results of the study were promising across four different hematologic malignancies with an area under the curves of 0.943 demonstrating high accuracy of the classification system. Also, these results can be achieved with only half of the FC markers (87).

Ni et al. investigated the use of support vector machine (SVM) to analyze MRD in AML patients using FC data. The SVM algorithm, adept in the realm of multidimensional analysis, can learn the features extracted from classified training data, create a model for recognition based on this information, and subsequently, classify obscure data with precision utilizing the established model. After using the optimal C and γ parameters, outcomes of SVM automated MRD analysis exhibited no significant difference when compared to the ones obtained from the manual method (P > 0.05). Additionally, the correlation coefficient between the two techniques was 0.986 (88). Hence the SVM model may be used to accurately analyze FC data collected from liquid biopsies from AML patients. However, further studies should further verify these findings as the sample size was small.

Ko et al. developed an AI algorithm by analyzing MFC data collected form BM aspiration of 1742 AML or MDS patients. The AI algorithm learned a multi-dimensional MFC phenotype from the training set and then input it to support vector machine classifier after Gaussian mixture model modeling. The AI algorithm was then applied to the validation set and results were compared. The average time spent analyzing one sample was only 7 sec compared to 20 min done manually by an experienced hematologist. This shows how AI can help reduce the manpower, time and training, needed to interpret MFCs. The results provided by the AI algorithm showed promising accuracy at 84.6% to 92.4% and area under the curve of 0.921-0.950. Also, AI analyzing a normal MFC of AML patients predicted better progression-free survival (p < 0.0001) and overall survival (p < 0.0001) (89). This study illustrates how AI can be used to efficiently analyze large sets of data. Presumably, the same concept could apply to MFC data taken from liquid biopsies.

As previously mentioned, LB is a noninvasive tool that could diagnose cancer but lacks standardization and adequate target characterization which in turn limits its application. Single cell RNA sequencing (scRNA-seq), a robust technology for cell characterization, can classify CTCs by their original lesions. However, its use is limited due to the shortage of CTC scRNA-seq data and prior information. Guo et al. designed a CTC-Tracer, a transfer learning-based algorithm that can transfer lesion labels from the primary cancer cell atlas to CTCs in order to correct the distributional shift between them. The purpose of the tracer is to trace back the lesion characterizing the original cancer by identifying gene expression of CTCs. They applied CTC-Tracer on a complex dataset consisting of RNA-seq profiles of single CTCs, CTC clusters from a BRCA patient, and two xenografts. Results showed high accuracy between 83.33% to 100% (90). Hence, CTC-Tracer has the potential to be applied to larger data sets of CTC as it has proved accuracy and efficiency in analyzing scRNA-seq data of different cancer types from various platforms. The introduction of CTC-tracer serves as an exciting opportunity to advance the use of liquid biopsy in both basic research and clinical applications (90).

A novel method for LBs relies on analyzing volatile organic compound (VOC) patterns in the blood headspace. An AI nanoarray was developed by compiling different sets of chemi-sensitive nano-based structured films to detect and stage cancer. To validate the nanoarray, breast, ovarian, and pancreatic cancer models were tested as they have shown high incidence and mortality rates in the population. The nanoarray has >84% accuracy, >81% sensitivity, and >80% specificity for early detection and >97% accuracy, 100% sensitivity, and >88% specificity for metastasis detection (91). Similar to other cancers, AML also exhibits metabolic changes that leads to producation of VOCs. Since VOC is reliant on metabolic activity, VOCs can be a great marker to identify MRD or those at risk of relapse (92). The above are a few examples where AI could play a role in LBs and MRD. As AI is still in the early stage of development and validation, more research is needed to validate its efficacy in clinical settings.

## 4 Conclusion

In summary, MRD assessment by LB is increasingly being recognized in myeloid malignancies as a powerful tool for prognostic and predictive purposes. Several clinical studies are incorporating blood based LBs using various modern techniques for MRD assessment as a correlative translational component. While high sensitivity flow cytometry is the most common technique for detection of circulating tumor cells in AML, next generation sequencing or RT-PCR can be used to identify a targetable mutation for surveillance of early relapse. Even though, MRD monitoring using LBs is not yet standard in myelodysplastic syndrome, this is an area of active investigation. As of now, in BCR/ABL negative MPN, LBs remain experimental research tools. Since the future of medicine is moving towards using artificial intelligence (AI), AI can potentially employ a useful algorithmic program to help solve the complexities of testing and interpretation in hopes to achieve recommendations for a personalized precision medical decision making algorithm. Current ongoing and future research will make this field more promising.

## Author contributions

SA and MB conceived the idea for the paper. SA, KN, FK, ZS,MK, SM, SV contributed to the writing of the manuscript. SA, MB, KN, FA, and CC reviewed and/or revised the manuscript before submission. All authors contributed to the article and approved the submitted version.

## Glossary

**Table:**

5-hydroxymethylcytosine	(5hmC)
Acute Myelocytic/myeloid Leukemia	(AML)
Acute Promyelocytic Leukemia	(APL)
Allogeneic stem cell transplantation	(alloSCT)
Anti-epithelial cell adhesion molecule	(anti-EpCAM)
Bone marrow	(BM)
Cell free DNA	(cfDNA)
Cell free RNA	(cfRNA)
Circulating endothelial cells	(CECs)
Circulating tumor cells	(CTCs)
Circulating tumor DNA	(ctDNA)
Circulating RNAs	(cRNAs)
Chronic Myelocytic Leukemia	(CML)
Clonal hematopoiesis of indeterminate potential	(CHIP)
Complete Response	(CR)
Deep learning	(DL)
Deep learning-based noninvasivecancer detection by integrating DNAsequence and methylation information of individual cell-free DNA reads	(DISMIR)
Digital droplet PCR	(ddPCR)
Discrimination of Rare EpiAlleles by Melt	(DREAMing)
Double drop-off ddPCR	(DDO-ddPCR)
Endothelial MPs	(EMPs)
Essential thrombocythemia	(ET)
European LeukemiaNet	(ELN)
Hematopoietic Stem Cell	HSC
International Society of liquid biopsy	(ISLB)
Leukemia-associated immunophenotypes	(LAIPs)
Liquid biopsy	(LB)
Micro RNAs	(miRNAs)
Microparticles	(MPs)
Minimal Residual Disease	(MRD)
Molecular chimerism	(MC)
Multiplanar flow cytometry	(MFC)
Multiparametric flow cytometry MRD	(FCM-MRD)
Myelodysplastic syndrome	(MDS)
Myeloproliferative Neoplasm	(MPN)
Next Generation Sequencing	(NGS)
Nanoparticle Tracking Analysis	(NTA)
Overall Survival	(OS)
Peripheral blood	(PB)
Peripheral blood mononuclear cells	(PBMC)
Platelet-derived MPs	(PMPs)
Polycythemia vera	(PV)
Primary myelofibrosis	(PMF)
Quantitative Reverse Transcriptase Polymerase Chain Reaction	(RT-qPCR)
Receiver Operating Characteristic	(ROC)
Red blood cell MPs	(RMPs)
Relapse free survival	(RFS)
Single cell RN	(scRNA)
Support Vector Machine	(SVM)
Tissue factor MPs	(TF + MPs)
Tumor educated platelets	(TEP)
Variant allelic frequency	(VAF)
Volatile Organic Compound	(VOC)
White blood cell	(WBC)
