# Supplementary material for: Liquid biopsies and minimal residual disease in myeloid malignancies
Source: Front Oncol. 2023 May 5;13:1164017. doi: 10.3389/fonc.2023.1164017 (PMC10196237; doi:10.3389/fonc.2023.1164017)
Supplement: Supplementary file 1 [file Table_1.docx]

Supplementary Material

**Liquid Biopsies and Minimal Residual Disease in Myeloid Malignancies**

**Sabine Allam^1^, Kristina Nasr^1^, Farhan Khalid^2^, Zunairah Shah^3^, Mahammed Ziauddin Khan Suheb^4^, Sana Mulla^5^, Sindhu Vikash^6^, Maroun Bou Zerdan^7^, Faiz Anwer^8^, Chakra P Chaulagain^9^**

^1^Department of Medicine and Medical Sciences, University of Balamand, Balamand, Lebanon

^2^Department of Internal Medicine, Monmouth Medical Center, New Jersey, USA

^3^Department of Internal Medicine, Weiss Memorial Hospital, Chicago, USA

^4^Department of Internal Medicine, St. Luke's hospital, Milwaukee, Wisconsin, USA

^5^Department of Internal Medicine, St Mary’s Medical Center, California, USA

^6^Jacobi Medical center/AECOM Bronx, NY, USA

^7^Department of Internal Medicine, SUNY Upstate Medical University, New York, USA

^8^Department of Hematology and Oncology, Taussig Cancer Center, Cleveland Clinic, Cleveland, OH, USA

^9^Department of Hematology and Oncology, Maroone Cancer Center, Cleveland Clinic Florida, Weston, FL, USA

*** Correspondence:**Corresponding Author
chaulac@ccf.org

# Supplementary Data

Table 1. Ongoing Studies Using/Studying the Utility of Liquid Biopsy in Myeloid Malignancies, provided by the ClinicalTrials.gov search engine, current as of the date of this article.

| **Clinical-**  **Trials.gov IDs** | **Title** | **Clinical Study Summary** | **Malignancy Under Study** | **Sample/**  **Biomarker** | **Technology Used** | **Ref.** |
| --- | --- | --- | --- | --- | --- | --- |
| NCT05366881 | cfMeDIP-seq Assay Multicenter Prospective Observational Validation for Early Cancer Detection, Minimal Residual Disease, and Relapse | Confirm genome-wide methylome enrichment platform to detect and differentiate amongst cancer types. Also, to detect minimal residual disease after completing cancer treatment and to detect relapse prior to clinical presentation in breast, colorectal, lung, and prostate cancer. | Brain,Breast, Bladder, Cervical, Colorectal, Endometrial, Esophageal, Gastric, Head and Neck, Hepatobiliary, Leukemia, Lung, Lymphoma, Multiple Myeloma, Ovarian, Pancreatic, Prostate, Renal, Sarcoma, and Thyroid | Blood Sample | Epigenomic-based genome-wide methylome enrichment platform | (1) |
| NCT05074316 | A Prospective Registry Study on Biological Disease Profile, Intervention Type and Clinical Outcome in Patients With Myeloid Neoplasms | Establish a registry study for patients with myeloid neoplasms by integrating clinical data, biological samples, socio-demographic information, long-term follow-up, and patient reported outcomes for scientific purposes. | All myeloid neoplasms | Blood and bone marrow samples, plasma and serum including liquid biopsies (cfDNA) | Comprehensive evaluation of the genomic, transcriptomic, epigenomic and proteomic "landscapes" and expressed surface antigens of myeloid disease | (2) |
| NCT00900224 | Assessment of Novel Molecular Markers in Acute Myeloid Leukemia | Study samples of tissue and blood from patients with cancer to identify DNA and biomarkers related to cancer. | AML | Bone marrow aspirate samples, blood and buccal cell samples, and bone marrow biopsy slides | PCR amplification, RT-PCR, and denaturing high-performance liquid chromatography. | (3) |
| NCT03138395 | Predicting Disease Relapse by Monitoring Circulating Cancer DNA After Chemotherapy in Patients with MDS and AML | Quantify and track peripheral blood plasma mutant allele frequency (MAF) in MDS and AML patients before, during and after chemotherapy treatment. Quantification of MAF from fingersticks and saliva samples to determine feasibility of obtaining adequate ctDNA for ddPCR. | AML and MDS | Peripheral blood sample, bone marrow biopsy, finger stick blood sample, saliva sample to obtain ctDNA | ddPCR | (4) |
| NCT01541800 | A Feasibility Study of Circulating microRNs as Disease Markers in Pediatric Cancers | Evaluate the presence of miRNAs in blood and cerebrospinal fluid of pediatric patients with central nervous system tumors, leukemia and lymphoma who are currently on chemotherapy. | Leukemia, Lymphoma, and Central Nervous System | Peripheral blood and  cerebrospinal fluid | Unspecified | (5) |
| NCT03702309 | Liquid Biopsy Evaluation and Repository Development at Princess Margaret | Develop an institution-wide Liquid Biopsy protocol to establish a common process for collecting blood and corresponding archived tumor specimens for future research studies as a means of non-invasively assessing tumor progression and response to treatment. | Lymphoma, Leukemia, other Solid tumors | Peripheral blood to collect circulating cfDNA, and cfRNA | Unspecified | (6) |
| NCT03496402 | Molecular and Immunological Characterization of High-Risk CHildhood Cancer at DiagnOsis, Treatment and Follow-up - Biological Evaluation in Children, Adolescents and Young Adults | Identify genetic alterations based on molecular characterization of tumor, characterize tumor microenvironment and the host's immunological profile, and compare between genetic variations identified at diagnosis to those on ctDNA during patient treatment, follow up, and/or relapse. | Leukemia, other Solid tumors | Peripheral blood, bone marrow, and cerebrospinal fluid to detect ctDNA | Unspecified | (7) |
| NCT03023202 | UWCCC Precision Medicine Molecular Tumor Board Registry | Evaluate the clinical utility of the Precision Medicine Molecular Tumor Board. It also aims to correlate mutations with protein overexpression, ctDNA, and spheroid culture investigations. | Solid and Hematological malignancy | Blood sample (to also detect ctDNA) and tissue sample | Unspecified | (8) |
| NCT01775072 | Genomic Profiling in Cancer Patients | Identify the nature and the frequency of “actionable”  oncogenic mutations to identify sensitive and resistance of tumors to FDA approved or investigational drugs. | Solid and Hematological malignancy | Tissue, blood, saliva, or nail clippings | Next generation Sequencing, RNA  sequencing | (9) |
| NCT01792882 | Prospective Collection of, Surplus Surgical Tumor, Tissues and Pre-surgical, Blood Samples | Collect specimens for a project that will identify genomic changes involved in human cancer. Specifically, the project will analyze DNA copy number changes, transcription profiles, epigenetic modifications, sequence variation, and sequence in both tumor tissue and case-matched germline DNA | Breast Cancer, Colorectal Cancer, Lung Cancer, Prostate Cancer, Hematologic Cancer | Surgical tumor tissues and presurgical blood samples | Unspecified | (10) |
| NCT01137643 | Tissue Procurement for Hematolymphoid Conditions | Provide source of patient tissue, blood, and body fluid samples for ongoing diagnostic, prognostic, or immune-monitoring studies. | Chronic Myeloproliferative Disorders, Leukemia, Lymphoma, Lymphoproliferative Disorder | Tissue, blood, and any bodily fluid | Unspecified | (11) |
| NCT02213822 | Molecular Testing of Cancer by Integrated Genomic, Transcriptomic, and Proteomic Analysis | Investigate genomic, transcriptomic, and proteomic alterations in human solid cancers and hematologic malignancies. | Solid tumors, Hematological Malignancies | Tissue, blood, and any bodily fluid | Next Generation Sequencing, exome sequencing, full-length transcriptome (mRNA) sequencing | (12) |
| NCT04298892 | Integrated Multiomics and Multilevel Characterization of Haematological Disorders and Malignancies | Identify mechanisms driving malignant disorders and transformation. Improve diagnosis and stratification of onco-hematological patients through associating biological and molecular features with patient’s clinical features identifying recurrence/MRD patterns after treatments, identifying prognostic and early diagnostic biomarkers, detecting circulating and tissue molecular markers while promoting technological advancement. | Hematological Cancers | Peripheral blood, bone marrow, biopsies | Unspecified | (13) |
| NCT04264767 | Characterization of Methylation Patterns in Cancer and Non-Cancer cfDNA, An Observational Multicenter Study | Identify methylation patterns that will discriminate cancer and noncancerous samples and the origin of cancer. | Solid and Hematologic malignancies | Blood Samples (to collect cfDNA), Urine Samples | Unspecified | (14) |
| NCT01772771 | Molecular Testing for the MD Anderson Cancer, Center Personalized, Cancer Therapy Program | Identify frequency and distribution of mutations and co-mutations between different tumor types to assist in personalized chemotherapy and create a database. As secondary endpoint, the study aims to identify circulating cfDNA to determine the concordance of specific alterations in the plasma and in tumor analysis, and to determine the change in cfDNA burden and mutation profile with treatment. | Solid and Hematologic malignancies | Tissue, blood, saliva/buccal swabs | Unspecified | (15) |
| NCT04290923 | Determination of Blood Tumor Cells | Assess if circulating tumor cells can be removed out of the blood with the help of magnetic nanoparticles coated with anti-EpCAM or anti-CD52 antibodies. | Prostate, Colon, Lung, Pancreatic cancer, Lymphoma, Leukemia | Blood sample | Unspecified | (16) |
| NCT02534649 | Bergonie Institute Profiling: Fighting Cancer by Matching Molecular Alterations and Drugs in Early Phase Trials | Identify actionable molecular alterations in cancer patients with advanced disease. | Solid Tumor, Hematologic malignancy | Biopsy, blood sample | NGS, immunological profiling | (17) |
| NCT00900198 | Tissue Procurement Protocol for the Developmental Therapeutics Clinic, National Cancer Institute (NCI) | Collect patient samples for research. | Lymphomas, Multiple Myeloma, Myelodysplastic Syndrome | Blood, serum, urine, tumor tissue, normal tissue, pleural fluid, CSF, saliva, bronchial alveolar lavage (BAL), circulating tumor cells, hair follicles, and bone marrow | Unspecified | (18) |

AML: Acute myeloid leukemia; MDS: myelodysplastic syndrome; cfDNA: cell free DNA; cfRNA: cell free RNA, ctDNA: circulating tumor DNA; ddPCR: Droplet digital PCR; miRNA: Micro RNA; anti-EpCAM: anti-epithelial cell adhesion molecule

1. cfMeDIP-seq Assay Prospective Observational Validation for Early Cancer Detection and Minimal Residual Disease. <https://ClinicalTrials.gov/show/NCT05366881>.

2. The Myeloid Neoplasms Biology and Outcome Project. <https://ClinicalTrials.gov/show/NCT05074316>.

3. Studying Tissue and Blood Samples From Patients With Acute Myeloid Leukemia. <https://ClinicalTrials.gov/show/NCT00900224>.

4. iCare3: Monitoring Circulating Cancer DNA After Chemotherapy in MDS and AML. <https://ClinicalTrials.gov/show/NCT03138395>.

5. Circulating microRNAs as Disease Markers in Pediatric Cancers. <https://ClinicalTrials.gov/show/NCT01541800>.

6. Liquid Biopsy Evaluation and Repository Development at Princess Margaret. <https://ClinicalTrials.gov/show/NCT03702309>.

7. Biological Characterisation of High Risk CHildhood Cancer in Children, Adolescents and Young Adults (MICCHADO). <https://ClinicalTrials.gov/show/NCT03496402>.

8. UWCCC Molecular Tumor Board Registry. <https://ClinicalTrials.gov/show/NCT03023202>.

9. Genomic Profiling in Cancer Patients. <https://ClinicalTrials.gov/show/NCT01775072>.

10. Prospective Collection of Surplus Surgical Tumor Tissues and Pre-surgical Blood Samples. <https://ClinicalTrials.gov/show/NCT01792882>.

11. Tissue, Blood, and Body Fluid Sample Collection From Patients With Hematologic Cancer. <https://ClinicalTrials.gov/show/NCT01137643>.

12. Molecular Testing of Cancer by Integrated Genomic, Transcriptomic, and Proteomic Analysis. <https://ClinicalTrials.gov/show/NCT02213822>.

13. Integrated Multiomics and Multilevel Characterization of Haematological Disorders and Malignancies. <https://ClinicalTrials.gov/show/NCT04298892>.

14. Characterization of Methylation Patterns in Cancer and Non-Cancer cfDNA. <https://ClinicalTrials.gov/show/NCT04264767>.

15. Molecular Testing for the MD Anderson Cancer Center Personalized Cancer Therapy Program. <https://ClinicalTrials.gov/show/NCT01772771>.

16. Determination of Blood Tumor Cells. <https://ClinicalTrials.gov/show/NCT04290923>.

17. Bergonie Institut Profiling : Fighting Cancer by Matching Molecular Alterations and Drugs in Early Phase Trials. <https://ClinicalTrials.gov/show/NCT02534649>.

18. Collection of Tissue Samples for Cancer Research. <https://ClinicalTrials.gov/show/NCT00900198>.
